# Optimization of lentiviral vector production using polyethylenimine-mediated transfection

**DOI:** 10.3892/ol.2014.2684

**Published:** 2014-11-07

**Authors:** YONG TANG, KENNETH GARSON, LI LI, BARBARA C. VANDERHYDEN

**Affiliations:** 1 Department of Urology, Affiliated Cancer Hospital of Guangxi Medical University, Nanning, Guangxi 530021, P.R. China; 2 Centre for Cancer Therapeutics, Ottawa Health Research Institute, University of Ottawa, Ottawa, Ontario K1H 8L6, Canada; 3 Department of Gynecologic Oncology, Affiliated Cancer Hospital of Guangxi Medical University, Nanning, Guangxi 530021, P.R. China; 4 Department of Cellular and Molecular Medicine, Centre for Cancer Therapeutics, Ottawa Health Research Institute, University of Ottawa, Ottawa, ON K1H 8L6, Canada

**Keywords:** lentivirus, vector production, polyethylenimine, calcium phosphate

## Abstract

The aim of the present study was to optimize the polyethylenimine (PEI)-mediated transfection method in order to simplify the efficient production of lentiviral vectors (LvVs), and to compare the CaPO_4_- and PEI-mediated transfection methods for producing LvVs. Different titration methods of LvV stocks, as well as different culture media, culture durations, cell densities and DNA quantities were compared to obtain an optimized procedure for the production of LvVs. Optimization of the production method for LvVs was achieved using PEI-mediated transient transfections. Serum-free Opti-MEM^®^ was used to directly produce LvVs that could be harvested 48 h after transfection. Furthermore, a cell density of 15×10^6^ cells/10-cm plate and a DNA concentration of 1X were selected for the optimum production of LvVs. The optimized LvV titration method was simple and direct; it involved LvVs carrying fluorescent reporters, which proved to be faster than the standard methods but equally as sensitive. In conclusion, a scalable process for production of LvVs by PEI-mediated transfection was established and optimized. The optimized PEI-mediated transfection method was easy to use, as well as providing greater reliability with a higher degree of reproducibility and consistency. Despite using less DNA, the PEI-mediated transfection method resulted in viral titers that were the same as those achieved using the CaPO_4_-mediated method.

## Introduction

Lentiviral vectors (LvVs) have emerged as an important tool for transgene delivery and gene therapy. LvVs offer various advantages for gene therapy due to their ability to infect quiescent, slowly dividing or non-dividing cells, such as hematopoietic stem cells, neurons and glial cells; their ability to integrate into the host cell genome, resulting in long-term transgene expression; and their large packaging capacity (1). Integrated LvVs do not induce an inflammatory or immune response, which allows long-term *in vivo* maintenance of transgene expression in a variety of tissue types.

Standard LvV production typically relies on the transient transfection of human embryonic kidney cells (HEK 293T) with a packaging plasmid, an envelope glycoprotein-encoding plasmid and a lentiviral transfer vector plasmid (2). Following transfection, lentiviral particles (LvPs) are produced and released into the culture supernatant of the HEK 293T cells. Although novel methods of producing of LvVs have been developed (3), and various transfection protocols using specific transfection reagents, such as Lipofectamine^®^ 2000 (4), SuperFect^®^ (5), FuGENE^®^ 6 and GeneJammer (6), have been successfully applied to the production of LvVs, high costs have hindered their extensive use. Thus, the transient transfection method using CaPO_4_ precipitation remains the most common method used for the production of LvVs (7). The main limitations of the CaPO_4_-mediated method of transient transfection are the large quantities of DNA required and the dependency of the method on an optimal pH of HEPES buffer. This pH can drift over time, resulting in large variability in transfection efficiency and significant batch-to-batch variation of the LvV titers (8). Furthermore, despite their safety and the marked progress that has been made in the development of lentiviral packaging systems, the average range of titers of crude lentiviral vector stocks produced is between 10^6^ and 10^8^ transduction units (TU) per ml (9–10). This range is useful for *in vitro* applications; however, the quantities are not high enough for *in vivo* use. For *in vivo* applications, large volumes of medium, large numbers of cells and large amounts of plasmid DNA are required to prepare sufficient stocks of crude LvVs. In addition, the relatively low transduction efficiency of LvVs means that the production of high-titer, high-quality LvV stocks is difficult and costly (11). Thus, improved methods for the consistent production of high-titer LvVs are required for the feasible use of LvVs in transgene delivery and gene therapy. Previously, the polyethylenimine (PEI)-mediated transient transfection method for LvV production was described (12); it uses a transfection reagent to produce recombinant viral vectors, such as adeno-associated viral vectors (AVVs) (13–14) or lentiviral vectors (12,15). There are various advantages of producing LvVs via the PEI-mediated transfection method. First, the relatively low cost of PEI allows it to be widely used in the research laboratory. Second, the chemical stability and pH independence of PEI ensures consistent high transfection efficiency (16–17). Finally, PEI has been demonstrated to be effective in transducing adherent and suspension cells (18–19).

The aim of the present study was to optimize the PEI-mediated transfection method in order to simplify the efficient production of LvVs, and to compare the CaPO_4_- and PEI-mediated transfection methods for producing LvVs. The PEI-mediated method was identified to be simple to use and more reliable, with a high degree of reproducibility and consistency. Despite using less DNA, the PEI-mediated transfection method resulted in viral titers that were the same as those achieved using the CaPO_4_-mediated method.

## Materials and methods

### Experimental cell lines

HEK 293T cells and murine fibroblastic NIH 3T3 cells were stocked in Dr Barbara C. Vanderhyden’s laboratory at the University of Ottawa (Ottawa, Ontario, Canada)and cultured in HyClone™ Dulbecco’s modified Eagle medium (DMEM)/high glucose (Thermo Fisher Scientific, Waltham, MA, USA) supplemented with 10% fetal bovine serum (FBS; GE Healthcare Bio-Sciences, Pittsburgh, PA, USA). The cells were maintained at 37°C and 5% CO_2_ in a humidified incubator (VWR International, LLC., Atlanta, GA, USA) and were harvested at 80–90% confluence.

### Plasmid constructs

The present study used a lentiviral transfer vector plasmid (pWPI) containing enhanced green fluorescent protein (eGFP), a pCMV-dR8.74 packaging plasmid and a pCAG4-Eco envelope plasmid, as previously described (20–21). pWPI and pCMV-dR8.74 plasmids were provided by Dr. D Trono (Swiss Federal Institute of Technology in Lausanne, Lausanne, Switzerland) and pCAG4-Eco plasmids were provided by Dr. A Nienhuis (St. Jude Children’s Research Hospital, Memphis, TN, USA).

### Preparation of PEI stocks

A 7.5 mM PEI stock solution was prepared by dissolving 0.15 g PEI (molecular weight, 40 kDa; Polysciences Inc., Warrington, PA, USA) in 245 ml tissue culture grade water. Once prepared, the PEI stock was stored at 4°C (22).

### PEI-mediated transfections were performed to generate lentiviral vectors

Lentiviral vectors were generated by the transient cotransfection of HEK 293T cells with a three-plasmid system. The lentiviral transfer vector plasmid (pWPI; 2 μg), packaging plasmid (pCMV-dR8.74; 1.3 μg) and envelope plasmid (pCAG4-Eco; 1.25 μg) DNA were mixed in 150 mM NaCl. Concurrently, 7.5 mM PEI stock was diluted 6-fold with 150 mM NaCl to yield a final PEI concentration of 1.25mM, using the formula previously described by Reed *et al* (13), and 0.38 ml was added to the 0.38 ml DNA (4.55 μg total) from above. Transfections were also performed using 2× DNA (9.10 μg) and 3× DNA (13.65) solutions. DNA/PEI mixtures were incubated at room temperature for ≥10 min. The HEK 293T cells were trypsinized, washed twice with 1X phosphate buffered saline (PBS) and resuspended (2×10^6^ cells/ml) in serum-free Opti-MEM^®^ (Invitrogen Life Technologies, Inc., Carlsbad, CA, USA). The DNA/PEI mixture was added to 7.5 ml suspended cells, and immediately plated onto a 10 cm and incubated at 37°C in a 5% CO_2_ humidified atmosphere. The supernatants containing LvVs were collected and stored at −80°C.

### CaPO_4_-mediated transfections were performed to generate LvVs

HEK 293T cells (2×10^6^ cells/well) were seeded in each 10 cm culture dish and incubated at 37°C in a 5% CO_2_ humidified atmosphere. After 24 h, transfer vector plasmid (pWPI; 20 μg), packaging plasmid (pCMV-dR8.74; 15 μg) and envelope plasmid (pCAG4-Eco; 6 μg) were diluted with 250 μl sterile double distilled water (Barnstead™ Nanopure™ ultrapure water purification system; Thermo Fisher Scientific, Inc.). An equal volume of 0.5 M CaCl_2_ was added. The DNA/CaCl_2_ mixture was added to 2X HBS (500 μl; 0.28 M NaCl, 0.05 M HEPES and 1.5 mM Na_2_HPO_4_; optimal pH range, 7.00–7.28) and incubated at room temperature for 30 min. Subsequently, the transfection mixture was applied to culture plates containing fresh culture media and incubated at 37°C in a 5% CO_2_ humidified atmosphere. After 48 h, the supernatants containing LvVs were collected and stored at −80°C.

### Optimal HBS pH was determined for the production of LvVs in HEK 293T cells

To empirically determine the optimal pH of HBS for use in the CaPO_4_-mediated transfections, trial experiments (pH range, 7.0–7.28) were conducted. Following the infection of NIH 3T3 cells (described below), the LvVs produced from the transfected cells was quantified using the direct titration method.

An appropriate number of NIH 3T3 cells were seeded in a culture plate and incubated at 37°C in a 5% CO_2_ humidified atmosphere. After 24 h, all of the culture medium was removed and virus medium containing polybrene was added to the well. The culture plate was centrifuged twice at room temperature and incubated at 37°C in a 5% CO_2_ humidified atmosphere.

### Titration of LvV stocks

#### Direct titration method

NIH 3T3 cells (2×10^5^ cells/ml) were trypsinized and resuspended in DMEM containing 10% FBS and polybrene was added to a final concentration of 8 μg/ml. The NIH 3T3 cells (1 ml) and viral supernatant (5–50 μl) were transferred into a 3 ml snap-top tube and mixed. The cell solution containing the virus was immediately seeded in individual wells of six-well plates and incubated at 37°C in a 5% CO_2_ humidified atmosphere. After 24 h, each well was supplemented with 1 ml fresh culture media. Three days after infection, the NIH 3T3 cells were trypsinized and the number of fluorescence-positive cells was determined using flow cytometry (Epics^®^ XL™ flow cytometer; Beckman Coulter, Inc., Mississauga, ON, Canada).

### Delayed titration method

NIH 3T3 cells (2×10^5^ cells/well) were seeded in six-well plates and incubated at 37°C in a 5% CO_2_ humidified atmosphere. After 24 h, the culture medium was removed and 1 ml fresh DMEM containing 10% FBS, 8 μg/ml polybrene and 5–50 μl viral supernatant were added to each well. After 24 h, each well was supplemented with 1 ml fresh culture medium. Three days after infection, the NIH 3T3 cells in each well were trypsinized and the number of fluorescence-positive cells was determined using flow cytometry (Epics XL flow cytometer; Beckman Coulter, Inc.).

### Titer formula

The following formula was used to convert the percentage of eGFP-expressing cells for a specific dilution into TU: TU/ml =[(% of cells expressing eGFP/100) × total number of NIH 3T3 cells at time of infection/volume of virus stock added (ml) (23).

### Flow cytometry

For immunostaining, cells were trypsinized and washed twice with 1X PBS. After determining cell number (Vi-Cell XR cell viability analyzer; Beckman Coulter, Inc.), 0.5–1×10^6^ cells were resuspended in 1 ml cold PBS or 100% formalin, protected from light and measured using an Epics XL flow cytometer (Beckman Coulter, Inc.) immediately or stored at 4°C for future use. A total of 1×10^4^–1×10^5^ events were analyzed in each sample and the resulting data were analyzed using Summit software (version 4.3; Dako North America, Inc., Carpinteria, CA, USA).

### Statistical analyses

Statistical analyses were performed using GraphPad Prism statistical software (GraphPad Software, Inc., La Jolla, CA, USA). Data was expressed as the mean ± standard error of the mean of three independent experiments performed in triplicate. The probability of significant differences between two groups or multiple groups was determined using Student’s t-test or analysis of variance, respectively. P<0.05 was considered to indicate a statistically significant difference.

## Results

### Optimization of lentivirus production

Although various commercial transfection reagents are available, the selection of a suitable transfection system was vital for the success of the present study. Virus production in serum-free conditions is ideal due to the possible future uses of virus purification and concentration. The key to successful LvV production is the efficient transfection of HEK 293T cells. For LvVs expressing fluorescent reporters, transfection efficiencies can be easily determined by the direct analysis of fluorescence in the transfected HEK 293T cells. Although more labor-intensive, the analysis of LvV production by viral titration using flow cytometry allows for a more precise determination of the optimal transfection method, choice of culture medium and timing of LvV harvest that cannot be adequately determined by looking solely at the transfection efficiencies of the HEK 293T cells. Therefore, prior to optimizing the transfection methods for the production of LvV, two titration methods were investigated.

### Comparison of different titration methods of LvV stocks

**Figure 1:**
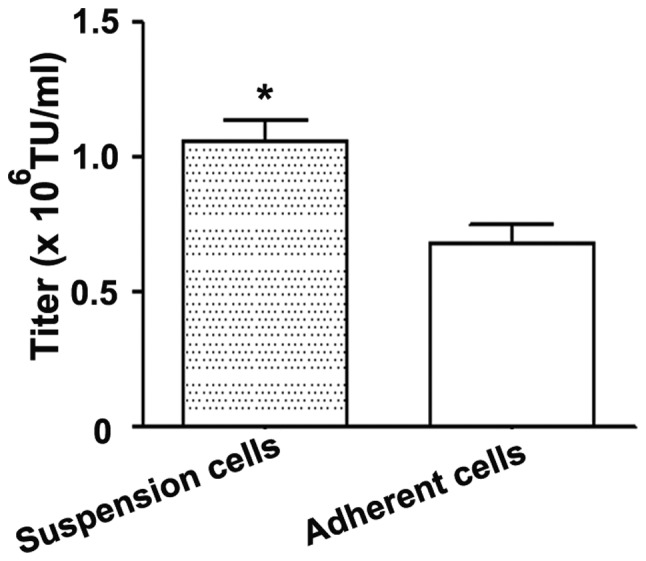
Comparison between the direct (suspension cells) and delayed (adherent cells) titration methods of LvV stocks. Titers of LvVs were determined 48 h after transduction of NIH3T3 cells. The calculated LvV titer was significantly higher for NIH3T3 cells infected in suspension when compared with cells infected while adhering to cell culture plates (n=3; ^*^P<0.001). LvV, lentiviral vector; TU, transduction units.

Two methods were evaluated for the determination of the LvV titer produced from the transfected HEK 293T cells. The first method (direct titration method) was performed as follows: Vector stock (5 μl) was immediately added to 1 ml culture medium containing 2×10^5^ freshly trypsinized NIH 3T3 cells suspension in a six-well plate. After 24 h, an additional 1 ml culture medium was added to each well. The second method (delayed titration method) was performed as follows: NIH 3T3 cells (2×10^5^) were plated in each well of a six-well plate. After 24 h, the media was removed and replaced with 1 ml culture medium containing 5 μl vector stock. After 24 h, an additional 1 ml culture medium was added to each well of the six-well plates. Three days after transduction of the NIH 3T3 cells, the percentage of eGFP-expressing NIH 3T3 cells was measured using flow cytometry and titers were calculated. As demonstrated by Fig. 1, the titer produced by direct titration was significantly higher than that obtained by delayed titration, which involved infected cells which had been plated 24 h earlier. These results indicated that the direct titration method, which is faster and simpler to perform, is a sensitive and convenient method for viral titration.

### Effect of different culture media and culture durations on the efficiency of PEI-mediated transfection

**Figure 2:**
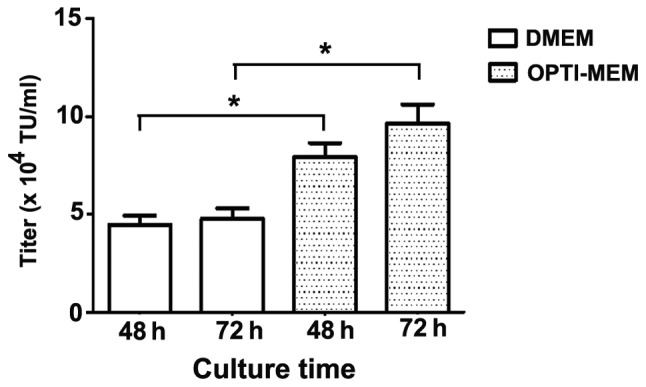
Effect of different culture media and times on the production of LvVs. Titers of LvV were obtained using polyethylenimine-mediated transient transfection with the human embryonic kidney 293T cells (cell density, 1×10^7^ cells/10-cm plate). The direct titration method was used and the dilution factor of lentivirus was 1:20. ^*^P<0.001. LvV, lentiviral vector; TU, transduction units; DMEM, Dulbecco’s modified Eagle medium.

In the initial production trial, HEK 293T cells were seeded at a density of 1×10^7^ cells/10-cm plate to produce LvVs. To quantify the amount of LvVs produced, the direct titration method was performed by adding 50 μl viral supernatant to each well containing NIH 3T3 cells. As demonstrated in Fig. 2, using serum-free Opti-MEM as the culture medium produced a higher titer compared with using DMEM, regardless of whether LvVs were harvested 48 or 72 h after transfection. Notably, the titers of LvV were not significantly different at 48 and 72 h post-transfection in the Opti-MEM or DMEM groups. These results indicated that serum-free Opti-MEM may be used directly to produce LvVs and that LvVs in the culture medium can be harvested 48 h after transfection, increasing the speed of the process.

### Influence of cell density on the efficiency of PEI-mediated transfection

**Figure 3:**
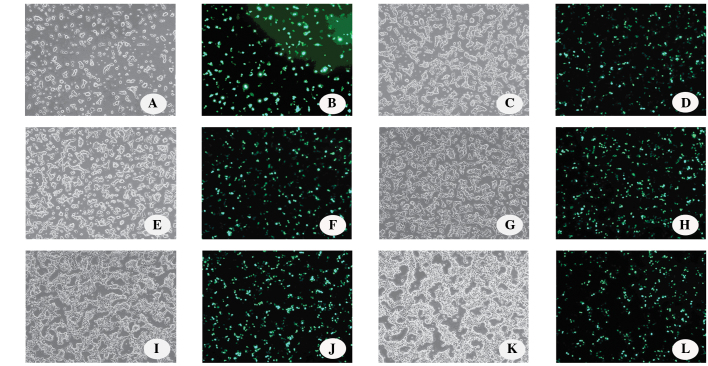
Effect of cell density on transfection efficiency in 10-cm plates was assessed by determining the number of green fluorescent protein-expressing cells by fluorescence microscopy (Axiovert 100 Fluorescence Microscope, Zeiss, Oberkochen, Germany; magnification, ×100). The cell densities evaluated were as follows: (A and B) 5×10^6^ cells/10-cm plate; (C and D) 10×10^6^ cells/10-cm plate; (E and F) 15×10^6^ cells/10-cm plate; (G and H) 20×10^6^ cells/10-cm plate; (I and J) 30×10^6^ cells/10-cm plate; and (K and L) 40×10^6^ cells/10-cm plate (magnification, ×100).

The optimal cell density for LvV production in 10-cm plates was investigated. Transient transfections were performed at low and high cell densities, from 5×10^6^ to 40×10^6^ cells/10-cm plate and the eGFP-expressing cells were observed under a fluorescent microscope (Fig. 3). Although the cells could achieve a density of >40×10^6^ cells/10-cm plate, the percentage of eGFP-expressing cells did not increase beyond this point when increasing the cell density during the transient transfection.

**Figure 4:**
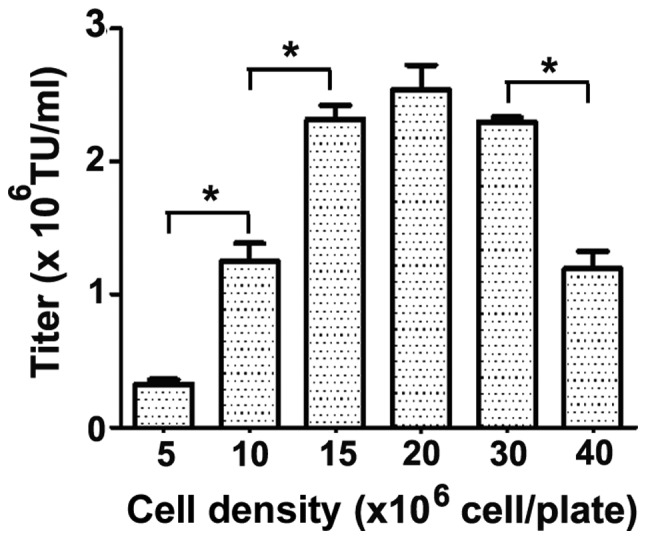
Optimization of cell densities for lentiviral vector production using 10-cm culture dishes. Experiments were conducted at low and high cell densities in serum-free Opti-MEM^®^. At densities of 5–15×10^6^ cells/10-cm plate, an increase in cell density resulted in higher viral titers at transient transfection. ^*^P<0.001. TU, transduction units.

As demonstrated in Fig. 4, vector titers were dependent on cell density and a significant increase in vector titers was achieved by increasing the cell density from 10×10^6^ cells/10-cm plate to 15×10^6^ cells/10-cm plate. However, increasing the cell density to ≥20×10^6^ cells/10-cm plate did not significantly increase the titer volume. Thus, 15×10^6^ cells/10-cm plate was selected as the optimum cell density for the production of LvVs in the subsequent experiments.

### Optimization of DNA quantity for PEI-mediated LvV production

**Figure 5:**
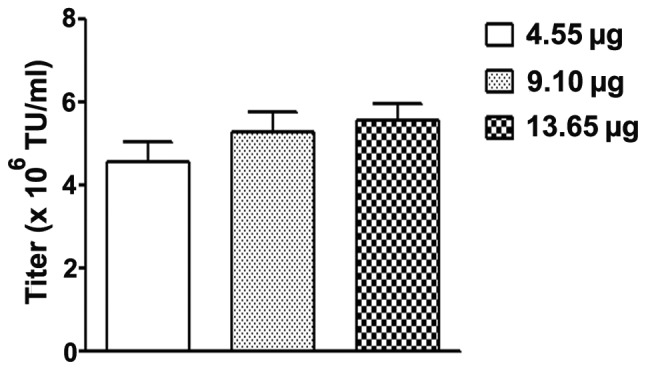
Optimization of DNA concentration for polyethylenimine-mediated LvV production in Opti-MEM^®^. LvVs were produced using 15×10^6^ HEK 293T cells per 10-cm plate and a total amount of 4.55 μg, 9.1 μg and 13.65 μg plasmid DNA mixture was added to 150 mm NaCl to a final volume of 380 μl, respectively. LvV, lentiviral vector; TU, transduction units.

Based on the results of the previous experiments, Opti-MEM medium and a HEK 293T cell density of 15×10^6^ cells/10-cm plate was selected to investigate the effects of altered DNA concentrations on the titers of LvV produced. The plasmid DNA solution (containing 4.55, 9.10 or 13.65 μg plasmid DNA) was prepared as described above. As demonstrated in Fig. 5, the LvV titer was not significantly different when different plasmid DNA concentrations were used. This indicates that the DNA concentration is not a key factor in the present experiment; thus, the DNA concentration could be standardized for future experiments (4.55 μg plasmid DNA mixture mixed with 150 mM NaCl; final volume, 380 μl).

### Optimization of the pH for LvV production using CaPO_4_

**Figure 6:**
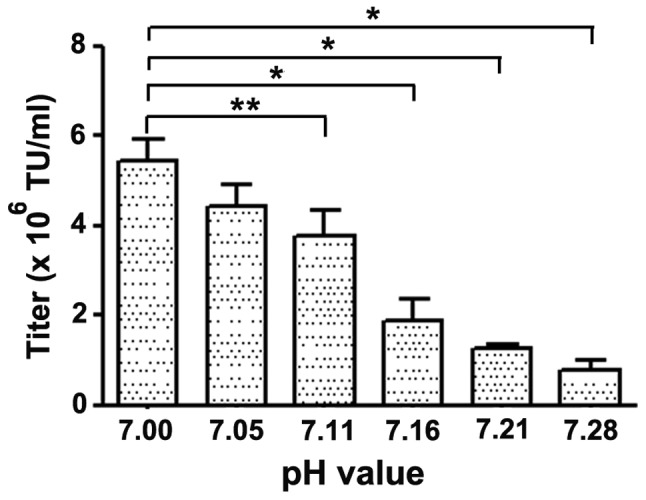
Optimization of the pH of the HBS buffer using the directed titration method. ^*^P<0.001 and ^**^P<0.05. TU, transduction units.

The pH of HBS was critical during CaPO_4_-mediated transient transfections. In the direct titration method, 7.00 and 7.05 were identified to be the optimum pH of HBS for CaPO_4_-mediated transient transfection (Fig. 6). These results indicated that the preferred HBS pH for LvV production using CaPO_4_ is 7.00. Both the direct observation of the percentage of eGFP-expressing HEK 293T cells by flow cytometry and the determination of LvV titers confirmed the optimal pH of HBS to be ~7.00. It was noted, however, that an HBS pH of 7.28 was effective at transfecting the HEK 293T cells based on direct flow cytometry, but produced a markedly lower LvV titer compared with at the optimal pH of 7.00.

### Comparison of LvV production using PEI- and CaPO_4_-mediated transient transfection

**Figure 7:**
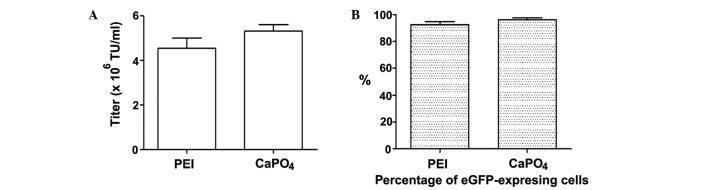
Comparison of lentiviral vector (LvV) production using PEI- and CaPO_4_-mediated transient transfection. (A) Comparison of the titers of LvV and (B) the percentage of eGFP-expressing cells 48 h after transfection produced by PEI-or CaPO_4_-mediated transfection in human embryonic kidney 293T cells. TU, transduction units; PEI, polyethylenimine; eGFP, enhanced green fluorescent protein.

The LvV titers produced by PEI- or CaPO_4_-mediated transfection were compared following the transfection of HEK 293T cells. As demonstrated in Fig. 7A, the LvV titers produced were not significantly different between the PEI- and CaPO_4_-mediated transfection methods.

The percentages of eGFP-expressing cells 48 h after transfection were determined by fluorescent microscopy observation of the culture plates or by analysis using a flow cytometer. No observable difference was identified in the proportion of eGFP-expressing cells between the PEI- and CaPO_4_-mediated transient transfection culture plates. Flow cytometry data confirmed that 92.25±2.80% of HEK 293FT cells transfected with PEI expressed eGFP, whereas 96.15±1.44% of the CaPO_4_-transfected cells were eGFP-positive. The percentages of eGFP fluorescent cells in the PEI- and CaPO_4_-transfected groups were not statistically different (Fig. 7B).

## Discussion

In the present study, the protocols for LvV production using PEI-mediated transient transfection were optimized. Vector titers were increased almost 100-fold after using these protocols and were similar to those achieved using CaPO_4_-mediated transient transfection. The protocol was simple, pH-independent, economic, reproducible and consistent.

Generally, two different methods are used to produce LvVs: The delayed titration method (which involves the development of stable vector packaging cell lines) and the transient titration method. Although several stable vector packaging cell lines have been generated (24), their use has various limitations. Firstly, the development of a stable vector packaging cell line requires a relatively long period of selection and characterization (25). Secondly, transgenes or vector components may carry toxicities that are not compatible with the development of a stable packaging cell line (26). Additionally, for each desired vector pseudotype, a new packaging cell line must be developed and, finally, stable packaging cell lines often produce relatively low vector titers and lack stability over prolonged culture periods, limiting their long-term applications (10). As a consequence, transient transfections of plasmids constitute a faster and simpler approach to LvV production and avoid the time-consuming development of stable packaging cell lines (27). In addition, various transgenes and envelope glycoproteins can easily be substituted in the transient transfection vector systems (28). Thus, the production of LvVs by transient transfection remains the most widely used technique and has been applied in the present study.

While the transient transfection method was used in all of the experiments in the present study, it was important to standardize a convenient system for the routine production of LvVs. PEI-mediated transient transfection, a simple and effective method, was investigated for the transient transfection of HEK 293T cells for the efficient production of LvV.

In the present study, various conditions, including the type of medium, harvest period, cell density, amount of plasmid DNA and titer method were evaluated and optimized to achieve the most favorable PEI-mediated transient transfection protocol. The resultant protocol significantly increased LvV titers by almost 100-fold compared with the non-optimized conditions. Furthermore, vector titers similar to those achieved using the CaPO_4_-mediated transient transfection method were obtained in the serum and protein supplement-free Opti-MEM medium. In addition to the advantages of using serum-free media, the PEI-mediated transfection method is simpler than the CaPO_4_ method in that the solutions are simple to prepare, not dependent on pH and transfected cells do not require their medium to be changed 24 h after transfection. Also, the PEI-mediated method requires approximately ten-fold less DNA to achieve similar amounts of LvV production to the CaPO_4_-mediated method. In the present study, PEI-mediated transient transfection demonstrated a high degree of reproducibility and consistency in its LvV production compared with CaPO_4_-mediated transfection. For example, the pH of the HBS used in the CaPO_4_-mediated method changes during storage, making it necessary to periodically re-prepare the buffer using the abovementioned empirical approach. Therefore, the use of the PEI-mediated transfection method simplifies the transfection procedure, significantly reduces the quantity of plasmid DNA and shortens the time required for LvV production.

Although the present study considered various factors during the optimization of PEI-mediated transient transfection for LvV production, there are numerous other parameters that could also be examined. For example, the ratio of PEI nitrogen to DNA phosphorous (N/P ratio) (29) and the quantity of DNA (22) have previously been demonstrated as important parameters for transient transfection. The PEI volume per plate used in the present study was calculated based on the protocol previously described by Kuroda *et al* (22). However, the PEI volume per plate may vary, perhaps in accordance with the equation described by Reed *et al* (13). The present study determined that 4.55 μg DNA mixture was adequate for the transfection of 1.5×10^7^ cells (0.3 μg DNA/10^6^ cells). This quantity is similar to that described in previous studies where 0.4–0.6 μg total DNA/10^6^ cells was used (22,30). Additional optimization of the amount of DNA used for PEI-mediated transfection could possibly be undertaken.

The use of different cell densities during PEI-mediated transient transfection resulted in significantly different titers (22) and the optimal cell density varied when different types of vectors were used. Based on the results obtained by Kuroda *et al* (22), LvV titers were relatively unaffected by 1–2×10^7^ HEK 293T cells/15-cm plate and the highest vector titers were obtained at a cell density of 1×10^7^ cells/15-cm plate. In the initial experiments of the present study, relatively lower titers were obtained when the HEK 293T cell density was 1×10^7^ cells/10-cm plate. This data confirms that the cell density is one of the defining conditions in optimizing the process of LvV production.

Furthermore, the present study did not evaluate the effect of the addition of nutrients on the efficiency of virus production. For example, the addition of peptones has been reported to lead to superior transfection efficiencies using PEI-mediated transfection (31–32).

The production of LvV in serum-free medium has previously been described (22). In two related studies, the PEI-mediated transfection method for LvV production was described, using Opti-MEM reduced serum medium (15) and serum-free, chemically defined (FreeStyle™) medium (12). By contrast, the present study used DMEM/high glucose and serum-free Opti-MEM reduced serum medium to directly culture HEK 293T cells. In previous studies, the LvV in the culture medium were typically harvested 48 h after transfection (13,22,33). Consistent with this, the present study identified that the optimal conditions of LvV production involved PEI-mediated transient transfection, serum-free medium and a 48-h incubation.

Although HEK 293T cells have been widely used for the production of LvV, including in the present study, HeLa cells and XDC293 cells have also been used as packaging cell lines for the production of recombinant viruses (13). In the present protocol, the titers of infective LvP were assessed by their ability to transfer the eGFP gene to the NIH 3T3 cell. This was similar to a study by Salmon and Trono (7), who recommended that the optimization of transfection protocols use a plasmid encoding GFP when establishing vector production procedures (7). Different LvV titration methods, including the determination of transgenic mRNA levels, correlate well with TU and can be used for the functional titration of non-fluorescent transgenes (34). Similarly, the quantification of LvV in the absence of fluorescent markers can be determined by quantitative polymerase chain reaction. The present study used NIH3T3 cells to titer LvV due to their restricted ability to infect only murine cells; however, HeLa cells have also been used as target cells for measuring titers of LvV pseudotyped with the vesicular stomatitis virus (VSV) G glycoprotein, allowing the infection of human cells (7). Although not all LvV titration methods were optimized, the present study provided a simple and efficient titration method for future use.

An additional consideration when using PEI-mediated transient transfection are the reported toxicities (30). The efficiency and cytotoxicity depend on the molecular weight of PEI. Smaller PEIs are non-cytotoxic but less efficient; for example, Toledo *et al* (33) obtained a high LvV titer (~1×10^7^ TU/ml) using branched 25 kDa PEI. Furthermore, cross-linked PEIs have been reported to enhance gene delivery efficiency (29). In the present study, which used 40 kDa PEI, no cytotoxicity was observed or evaluated; however, the vector titers were good enough for use in future transduction experiments.

For the optimization of CaPO_4_-mediated transient transfection, only the pH of HBS was optimized, as other parameters had previously been optimized in the laboratory. However, the titer of CaPO_4_-mediated transient transfection was affected by the structure of the envelope plasmid; the titer obtained from the envelope protein of VSV G glycoprotein was ~10-fold higher than from the envelope protein of rabies G glycoprotein (35). In addition, the titer of CaPO_4_-mediated transient transfection was affected by the concentration of CaPO_4_, the time of complex formation (36) and the cell cycle (37–38).

In the present study, similar titers of LvV were produced from PEI- and CaPO_4_-mediated transient transfection. Similarly, although Toledo *et al* (33) obtained a high LvV titer using PEI, the PEI-mediated transfection method did not demonstrate a higher titer compared with the CaPO_4_-mediated transfection method. By contrast, for the production of recombinant AVVs in HEK293 and HeLa cells, linear PEI was a better transfection reagent than CaPO_4_ (13). The vector titers achieved by transient transfection in the present study were relatively low compared with the typical titers achieved in previous reports (10^6^–10^7^ TU/ml) (22,30). This may have been due to the choice, for biosafety considerations, to pseudotype the LvV with the ecotropic envelope protein. The use of this envelope protein has consistently been demonstrated to result in lowered titers when compared with the VSV G glycoprotein (22,33).

Although numerous other factors may be optimized to improve LvV production, it is apparent that the PEI-mediated transfection method is the most cost-effective option (19). In addition to improved transfection efficiencies, LvV titers may be improved by the processing of cell culture supernatants to purify and concentrate the virus using serum-free media (22), physical concentration (11), ultracentrifugation (28), HYPERFlask^®^ vessels (39) or filtration approaches (40), such as diafiltration (15). Although the LvV produced in the present study was not purified, the use of the PEI-mediated transfection method and protein-free media should allow this route to be pursued if required.

In conclusion, the present study developed, described and optimized a scalable process for the production of LvV by PEI-mediated transfection. Interest in the use of LvV for human gene therapy is high and it has even undergone phase I clinical trials to investigate its possible use in the treatment of human immunodeficiency virus (41). However, large-scale LvV production remains a challenge for clinical applications. Thus, additional developments in LvV production and purification strategies will be essential to improve the cost-effectiveness of LvV use in clinical studies.
